# The chloroplast genome of *Cerasus humilis*: Genomic characterization and phylogenetic analysis

**DOI:** 10.1371/journal.pone.0196473

**Published:** 2018-04-25

**Authors:** Xiaopeng Mu, Pengfei Wang, Junjie Du, Yu Gary Gao, Jiancheng Zhang

**Affiliations:** 1 College of Horticulture, Shanxi Agricultural University, Jinzhong, Shanxi, P. R. China; 2 Shanxi Key Laboratory of Germplasm Improvement and Utilization in Pomology, Jinzhong, Shanxi, P. R. China; 3 OSU South Centers, College of Food, Agricultural, and Environmental Sciences, The Ohio State University, Shyville Road, Piketon, Ohio, United States of America; International Centre for Genetic Engineering and Biotechnology, INDIA

## Abstract

*Cerasus humilis* is endemic to China and is a new fruit tree species with economic and environmental benefits, with potential developmental and utilization applications. We report the first complete chloroplast genome sequence of *C*. *humilis*. Its genome is 158,084 bp in size, and the overall GC content is 36.8%. An inverted repeats (IR) of 52,672 bp in size is separated by a large single-copy (LSC) region of 86,374 bp and a small single-copy (SSC) region of 19,038 bp. The chloroplast genome of *C*. *humilis* contains 131 genes including 90 protein-coding genes, 33 transfer RNA genes, and 8 ribosomal RNA genes. The genome has a total 510 simple sequence repeats (SSRs). Of these, 306, 149, and 55 were found in the LSC, IR, and SSC regions, respectively. In addition, a comparison of the boundaries of the LSC, SSC, and IR regions of ten other *Prunus* species exhibited an overall high degree of sequence similarity, with slight variations in the IR boundary region which included gene deletions, insertions, expansions, and contractions. *C*. *humilis* lost the *ycf1* gene at the IRA/SSC border and it has the largest *ycf1* gene at the IRB/SSC border among these *Prunus* species, whereas the *rps19* gene was inserted at the IRB/LSC junction. Furthermore, phylogenetic reconstruction using 61 conserved coding-protein genes clustered *C*. *humilis* with *Prunus tomentosa*. Thus, the complete chloroplast genome sequence of *C*. *humilis* provides a rich source of genetic information for studies on *Prunus* taxonomy, phylogeny, and evolution, as well as lays the foundation for further development and utilization of *C*. *humilis*.

## Introduction

*Cerasus humilis* (Bge.) Sok is a bush fruit tree that is endemic to China; its fruits are rich in calcium and are thus also known as “Calcium fruit” [1]. It is mainly distributed in the northeast, northwest, north, and other northern areas of China [2]. *C*. *humilis* has long existed in the wild, and studies on this species were only initiated in the 1990s [3]. After nearly 30 years of research studies, identification of varieties, and establishment of cultivation techniques, our understanding of this fruit tree species has improved and facilitated its large-scale artificial cultivation. It has a strong root system and it shows strong adaptability to saline soil, harsh winter and drought [4]. Furthermore, it has strong soil erosion resistance, therefore it can be used for the improvement of damaged soil and environmental greening [5]. *C*. *humilis* is also used as an ornamental plant, generating striking white or red flowers. Its fruits look like cherries but have various colors including red, yellow, white, green, purple, etc. Moreover, it is a high-yield crop and its fruit has a unique flavor. The fruits can be eaten fresh, preserved, or processed into wine or other by-products. Its stems can be used in weaving and as high-protein cattle feed. The seed kernels have been traditionally used in Chinese medicine as “Yuliren” [5,6]. Based on its ecological and economic benefits, *C*. *humilis* has become an emerging multi-purpose fruit trees with a broad developmental and utilization potential.

Similar to the mitochondria and nuclei, chloroplasts are semi-autonomous organelles that function in the transfer and expression of genetic material DNA [7,8]. Chloroplasts are generally situated in the cytoplasmic matrix and play an important role in photosynthesis and fatty acid, starch, and amino acid synthesis [9]. In 1986, the complete chloroplast genome sequence of *Marchantia polymorpha* and tobacco (*Nicotiana tabacum*) were released and to date, the chloroplast whole genome sequence of 700 plant species has been completed [10]. The chloroplast genome is a closed cyclic structure that consists of large single copy (LSC), small single copy (SSC), and two reverse repeats (IR); its genome size ranges from 100 kb to 200 kb. Protein-coding genes are important in photosynthesis and other functions of the chloroplast and are further divided into nine categories: Photosystem I, cytochrome b6-f complex, Photosystem II, ATP synthase, ribulose bis-phosphate carboxylase, ribosome large subunit, ribosome small subunit, RNA polymerase, and a hypothetical protein [11]. The overall structure and genome of the chloroplast are relatively conserved, although in-depth studies in chloroplast genomics and biology have identified several mutations and small structural changes among various species including insertions, deletions, reversals, and translocations [12,13].

A famous Chinese fruit scientist earlier classified *C*. *humilis* as *Rosaceae cerasus* [14], whereas peach, apricot, plum, and cherry are classified as *Prunus*. Although *Prunus* consists of numerous species, only the chloroplast genome of ten species including *Prunus kansuensis*, *Prunus maximowiczii*, *Prunus pseudocerasus*, *Prunus yedoensis*, *Prunus persica*, *Prunus tomentosa*, *Prunus serotina*, *Prunus dulcis*, *Prunus cerasoides* and *Prunus padus* have been completely sequenced to date, with genome sizes ranging from 157,685 bp–158,955 bp. *C*. *humilis* may be further developed and potentially utilized in various industries. The complete chloroplast genome sequence of *C*. *humilis* could be applied in facilitating evolutionary analysis, accelerating breeding, identification of commercial cultivars and determination of varieties’ purity. In this study, the whole genome sequence of the chloroplast of *C*. *humilis* was obtained for the first time, and its structural characteristics and phylogenetic relationships were analyzed.

## Materials and methods

### Plant material and DNA extraction

**Fig 1:**
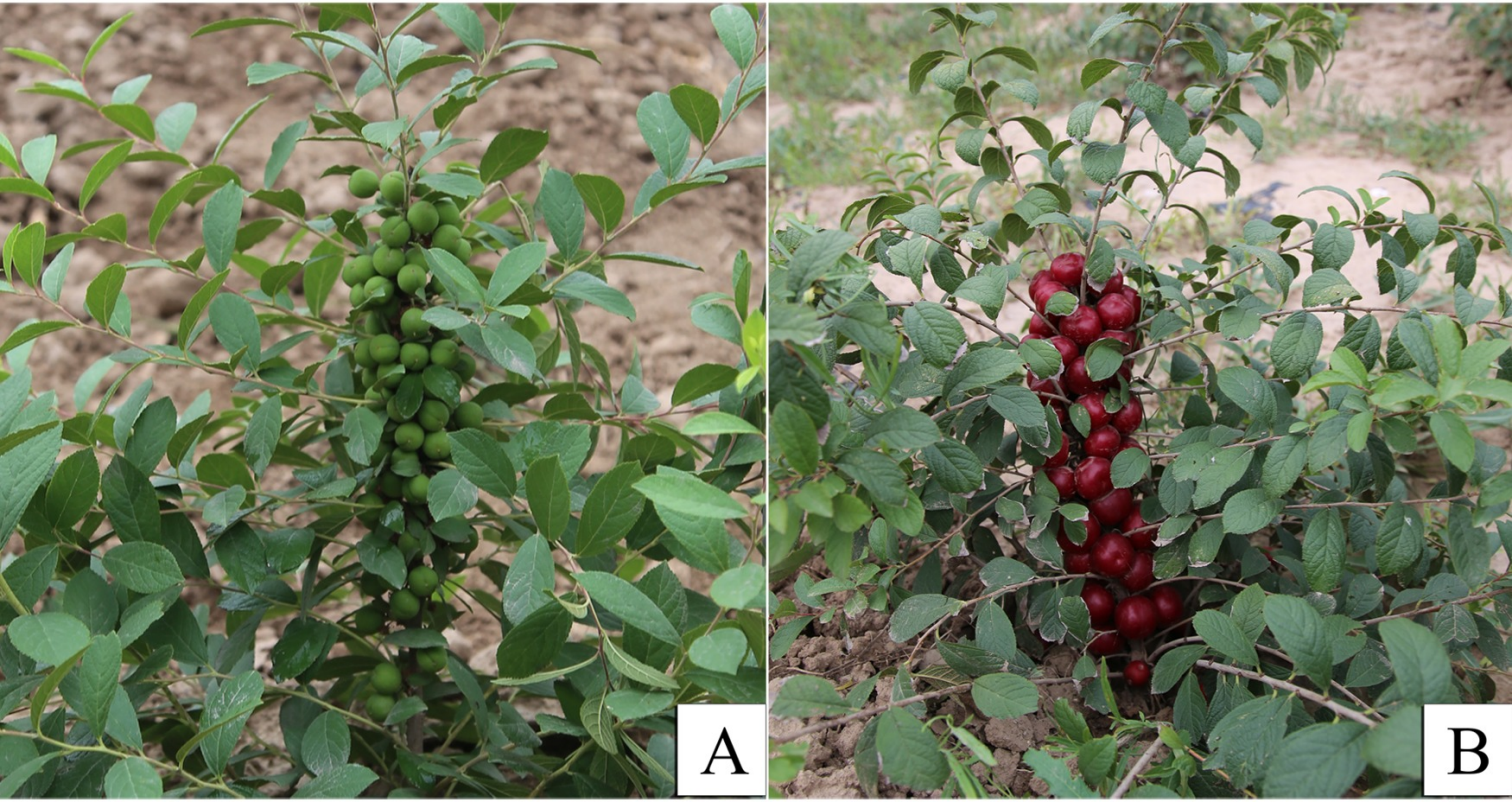
Whole plant of *C*. *humilis* (cv. ‘Nongda 4’). (A) Young fruit stage. (B) Mature fruit stage.

Fresh and young leaves of the upper part of the branches from a cultivated variety of *C*. *humilis* (cv. Nongda 4, Fig 1) were collected from the germplasm resources of *C*. *humilis* of Shanxi Agricultural University (37°26'N, 112°32'E). Using 100 mg of a fresh plant sample, total genomic DNA was isolated using HP Plant DNA Kit D2485-01 (Omega Bio-Tek, Santa Clara, CA, USA), according to the manufacturer’s protocol.

### Chloroplast genome sequencing, assembly, and annotation

After quantification and qualification of total genomic DNA of the sample, a paired-end library of 2 × 150 bp and insert size of ~350 bp was constructed according to the Illumina standard library, which was then sequenced on an Illumina Hiseq 2500 platform. The resulting low-quality raw data were filtered, and the linker sequences were removed, thereby resulting in 20.86 Gb of clean data, with a quality value ≥ Q30, accounting for 91.37%. The genome of *P*. *pseudocerasus* was used as reference (GenBank Accession No. NC_030599.1), and NOVOPlasty software was used to assemble the chloroplast genome, the mistake parameter was set by default [15]. Three software were used for genome degenerate base correction, namely, bwa, samtools, and bcftools [16,17]. The complete *C*. *humilis* chloroplast genome sequence was annotated using the online software CpGAVAS developed by Liu et al [18] (http://www.herbalgenomics.org/0506/cpgavas/analyzer/annotate). TRNAscan-SE was used in tRNA confirmation [19]; http://lowelab.ucsc.edu/tRNAscan-SE/). Genomic circle graphs were drawn using OGDRAW [20] (http://ogdraw.mpimp-golm.mpg.de/). The complete *C*. *humilis* chloroplast genome was submitted to GenBank, as Accession Number MF405921.

### Comparative genome analysis

To investigate the differences between the *C*. *humilis* genome sequence and those of the other members of the same genus, we used the LAGAN alignment strategy as implemented in the mVISTA software to compare the whole genome sequence of ten species of *Prunus* with *C*. *humilis*, using *C*. *humilis* as reference [21] (http://genome.lbl.gov/cgi-bin/VistaInput?num_seqs=2). In addition, the differences in the types and gene sizes of the IR border genes of five species were analyzed.

### Simple sequence repeat (SSR) analysis

Microsatellites are also known as SSRs, which occur as mono-, di-, tri-, tetra-, penta-, and hexa-nucleotide repeats. The SSRs in the chloroplast genome of *C*. *humilis* were identified using PHOBOS 3.3.12, and the alignment was screened in terms of matches, mismatches, gaps, and N positions using the scores of 1, -5, -5, and 0, respectively [22]. At the same time, the SSR of the IR, LSC, SSC, and coding regions, introns, and intergenic regions corresponding to different regions were analyzed.

### Phylogenetic analysis

The chloroplast genome sequences of ten *Prunus* species were downloaded from NCBI. Approximately 61 coding-protein genes were identified in *C*. *humilis* and the other ten *Prunus* species, which were then aligned using mafft software's FFT-NS-2 algorithm [23]. After the results were integrated, phylogenetic reconstruction was performed using RAxML Version 8 tool as implemented in CIPRES [24].

## Results

### General features of the *C*. *humilis* chloroplast genome

**Fig 2:**
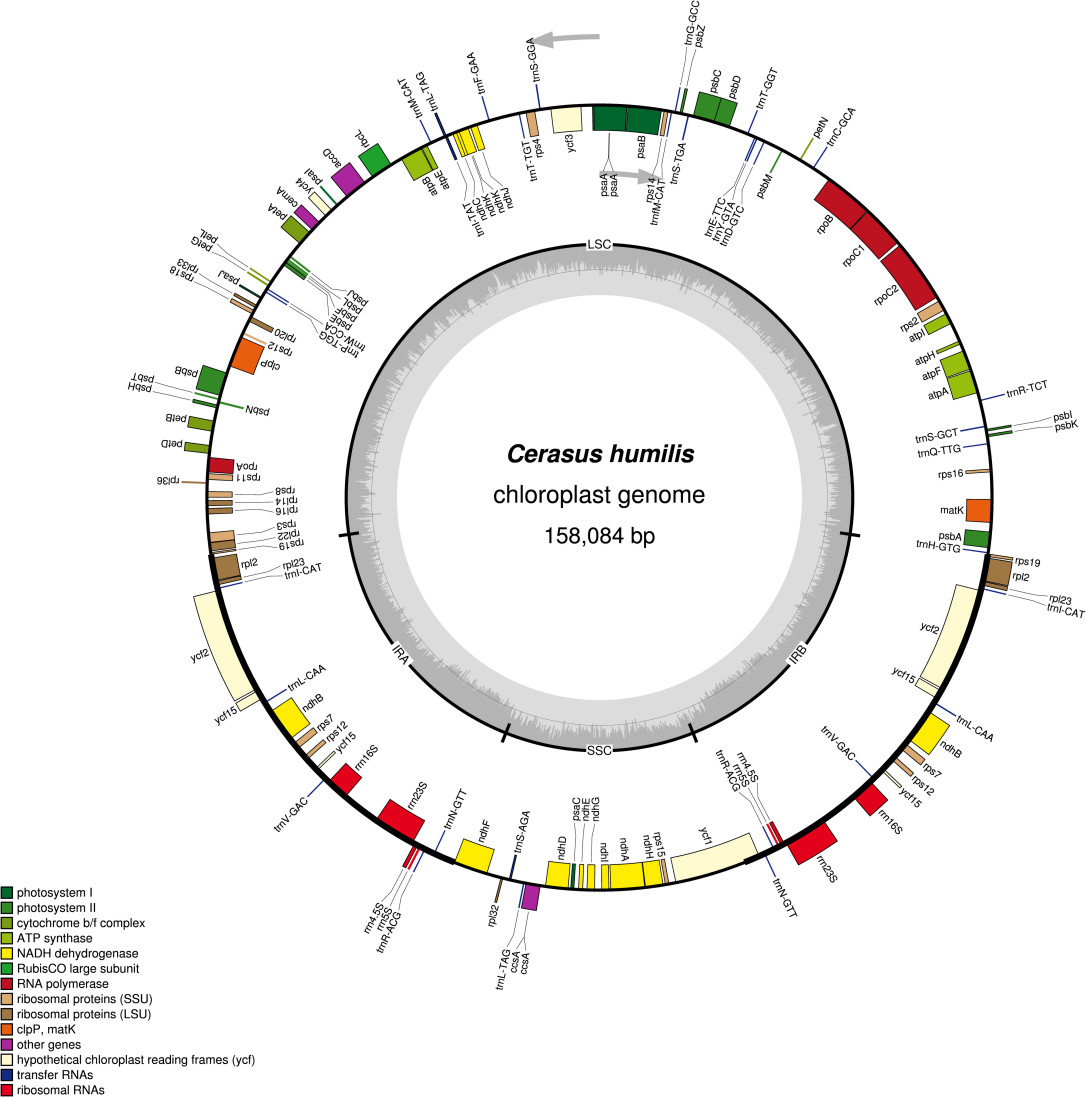
Gene map of the *C*. *humilis* chloroplast genome. Genes drawn inside the circle are transcribed clockwise, whereas genes outside are transcribed counterclockwise. Genes are color coded by their function in the legend. The area in darker gray and lighter gray in the inner circle indicates GC content and AT content, respectively.

The genome of the *C*. *humilis* circular chloroplast is 158,084 bp in size and exhibits the typical quadripartite structure found in most land plants (Fig 2). The IR region spans 52,672 bp, whereas the large single copy (LSC) and small single copy (SSC) region span 86,374 bp and 19,038 bp, respectively. GC contents of the complete genome is 36.8%, the LSC and SSC region are 34.5% and 30.5%, respectively, whereas that of the IR regions is 42.6% (Table 1). Similar to other terrestrial plants such as *Cynara humilis*, a species of family Asteraceae, the IRS showed higher GC content, which was mainly due to the rRNA genes *rrn16*, *rrn23*, *rrn4*.*5*, and *rrn5* [25].

**Table 1:** Summary statistics for assembly of *C*. *humilis* chloroplast genomes.

Genome features	*Cerasus humilis*
Size (bp)	158,084
LSC length (bp)	86,374
SSC length (bp)	19,038
IR length (bp)	52,672
Number of gene	131
Protein-coding genes	90
tRNA genes	33
rRNA genes	8
GC content (%)	36.8
GC content in LSC (%)	34.5
GC content in SSC (%)	30.5
GC content in IR (%)	42.6

There are a total of 131 genes, including 90 protein-coding genes, 33 transfer RNA (tRNA), and 8 ribosomal RNA (rRNA) genes. Among these, 17 genes have two copies while *rps15* and *rps12* have more than copies (Tables 1 and 2). In the functional classification of genes, 44 genes (unique) are associated with photosynthetic functions such as *atpH*, *ndhD*, and *rbcL*. The function of the conserved open reading frames of *ycf* (*ycf1*, *ycf2*, *ycf3*, *ycf4* and *ycf15*) is unknown. The nine genes, namely, *atpF*, *ndhA*, *ndhB*, *petB*, *rpl16*, *rpl2*, *rpoC1*, *rps16*, and *ycf1*, contain a single intron. The two copies of the *rpl2* and *ndhB* genes all have one intron. In addition, there are two introns in the *ycf3* and c*lpP* genes (Table 2).

**Table 2:** Gene content in *C*. *humilis* chloroplast genome.

Function	Family	Genes
**Genes for photosynthesis**	Subunits of ATP synthase	*atpA*, *atpB*, *atpE*, *atpF* ^*a*^, *atpH*, *atpI*
	Subunits of NADH-dehydrogenase	*ndhA* ^a^, *ndhB* ^a^ (2), *ndhC*, *ndhD*, *ndhE*, *ndhF*, *ndhG*, *ndhH*, *ndhI*, *ndhJ*, *ndhK*
	Subunits of cytochrome b/f complex	*petA*, *petB* ^a^ *petD*, *petG*, *petL*, *petN*
	Subunits of photosystem I	*psaA*, *psaB*, *psaC*, *psaI*, *psaJ*
	Subunits of photosystem II	*psbA*, *psbB*, *psbC*, *psbD*, *psbE*, *psbF*, *psbH*, *psbI*, *psbJ*, *psbK*, *psbL*, *psbM*, *psbN*, *psbT*, *psbZ*
	Subunit of RuBisCO	*rbcL*
**Other genes**	Subunit of Acetyl-CoA-carboxylase	*accD*
	c-type cytochrome synthesis gene	*ccsA*
	Envelop membrane protein	*cemA*
	Protease	*clpP* ^b^
	Maturase	*matK*
**Self-replication**	Large subunit of ribosome	*rpl14*, *rpl16* ^a^, *rpl20*, *rpl22*, *rpl23* (2), *rpl2* ^a^ (2), *rpl32*, *rpl33*, *rpl36*
	DNA-dependent RNA polymerase	*rpoA*, *rpoB*, *rpoC1* ^a^, *rpoC2*
	Small subunit of ribosome	*rps11*, *rps12* (3), *rps14*, *rps15*, *rps16* ^a^, *rps18*, *rps19* (2), *rps2*, *rps3*, *rps4*, *rps7* (2), *rps8*
	rRNA Genes	*rrn4*.*5S* (2), *rrn5S* (2), *rrn23S* (2), *rrn16S* (2)
	tRNA genes	*trnR-ACG* (2), *trnS-TGA*, *trnT-TGT*, *trnS-GCT*, *trnF-GAA*, *trnH-GTG*, *trnD-GTC*, *trnL-CAA* (2), *trnE-TTC*, *trnG-GCC*, *trnI-CAT* (2), *trnI-TAT* (2), *trnR-TCT*, *trnL-TAG*, *trnM-CAT*, *trnY-GTA*, *trnQ-TTG*, *trnT-GGT*, *trnS-AGA* (2), *trnN-GTT* (2), *trnW-CCA*, *trnfM-CAT*, *trnS-GGA*, *trnP-TGG*, *trnV-GAC* (2), *trnC-GCA*
**Unknown function**	Conserved open reading frames	*ycf1*^a^, *ycf2* (2), *ycf3*^b^, *ycf4*, *ycf15* (4)

^a^: Gene containing a single intron

^b^: Gene containing two introns.

### Comparative genome analysis

**Fig 3:**
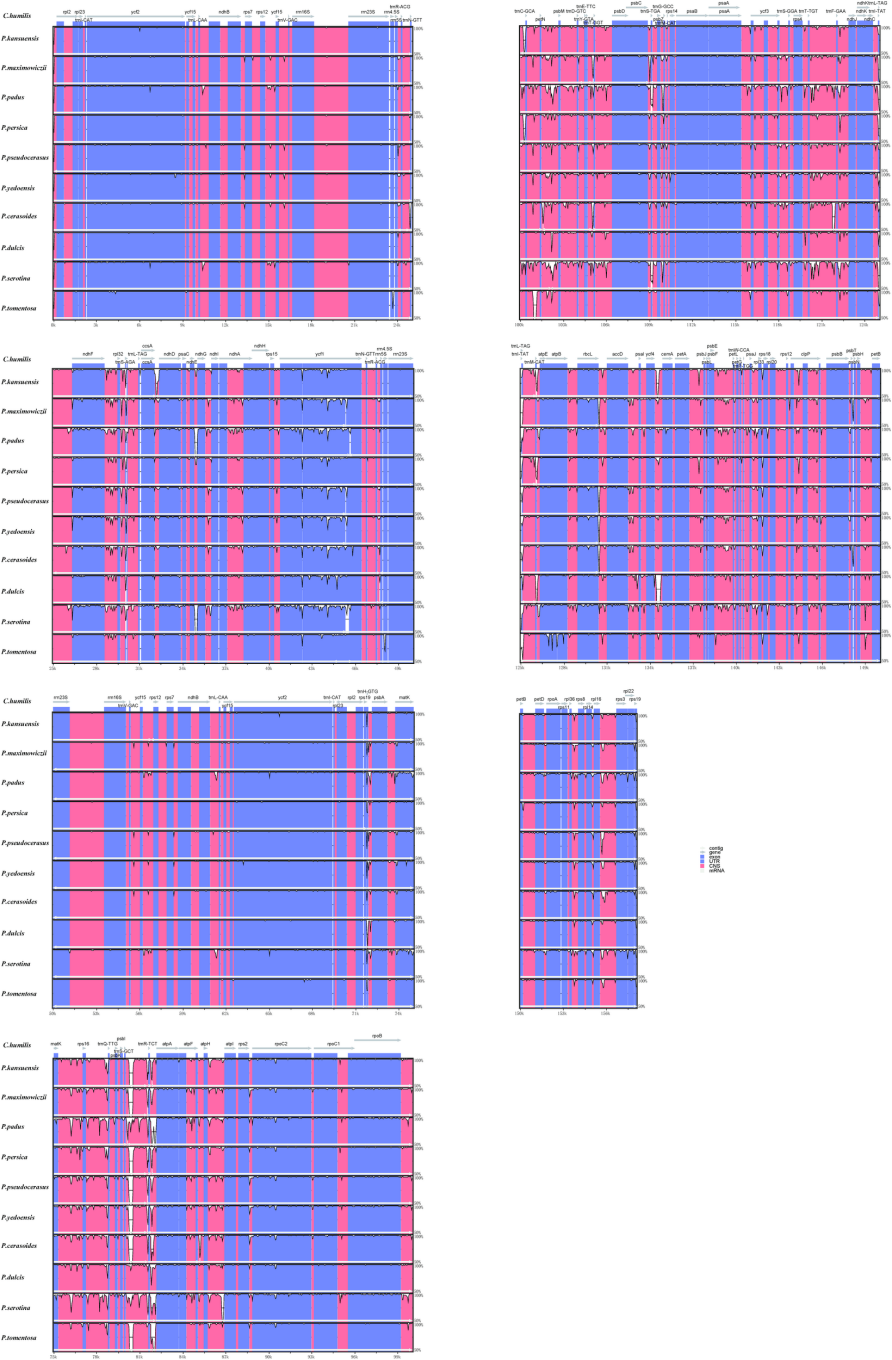
Visualization of genome alignment of the chloroplast genomes of ten *Prunus* species using *C*. *humilis* as reference using mVISTA. Vertical scale indicates the percentage of identity, which ranges from 50% to 100%. Coding regions are marked in blue, and non-coding regions are indicated in red. Gray arrows indicate the position and direction of each gene.

To further analyze the characteristics of the *C*. *humilis* chloroplast genome, we compared the assembled genomes with the chloroplast genomes of ten other *Prunus* species in NCBI. First, we compared the basic characteristics of their genomes. The size of the *C*. *humilis* chloroplast genome is smaller than those of *P*. *padus, P. pseudocerasus* and *P*. *tomentosa*. However, it has the highest GC content and the second largest number of encoded proteins (N = 90). In addition, it has 131 genes in total and the number of tRNA genes is the lowest among these 11 members of the *Prunus* genus (Table 3). The sequence identity was analysed using program mVISTA by aligning the ten *Prunus* species’ chloroplast genomes with *C*. *humilis *(Fig 3). As expected, the sequence identity of the eleven chloroplast genome sequences was high, indicating that these are highly conserved. However, several variations were found in noncoding and single copy regions of these eleven chloroplast genome (Fig 3).

**Table 3:** Comparison of chloroplast genome characteristics in various *Prunus* species.

Species	GenBank Acc. No.	Size (kb)	GC%	Protein	rRNA	tRNA	Gene	Pseudogene
*Cerasus humilis*	MF405921	158.084	36.80	90	8	33	131	-
*Prunus kansuensis*	NC_023956.1	157.736	36.76	86	8	39	133	-
*Prunus maximowiczii*	NC_026981.1	157.852	36.73	84	8	37	129	-
*Prunus persica*	NC_014697.1	157.79	36.76	85	8	37	131	1
*Prunus pseudocerasus*	NC_030599.1	157.834	36.73	86	8	37	131	-
*Prunus yedoensis*	NC_026980.1	157.859	36.73	84	8	37	129	-
*Prunus serotina*	NC_036133.1	158,778	36.60	82	8	36	126	-
*Prunus dulcis*	NC_034696.1	157,723	36.80	86	0	38	124	-
*Prunus cerasoides*	NC_035891.1	157,685	36.70	84	8	37	129	-
*Prunus tomentosa*	NC_036394.1	158,356	36.80	91	0	36	127	-
*Prunus padus*	NC_026982.1	158.955	36.59	84	8	37	129	-

**Fig 4:**
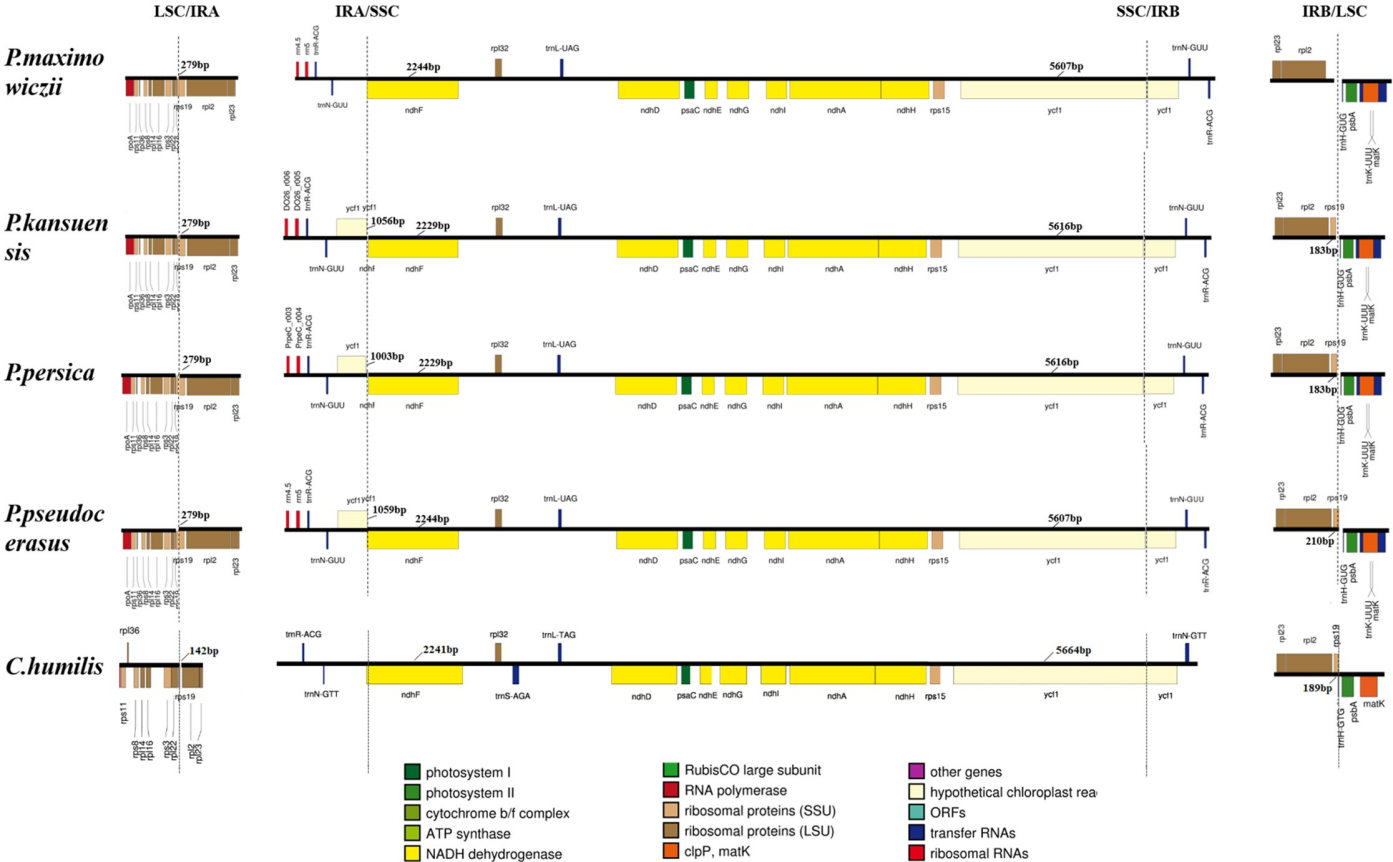
Comparison of the boundaries of the LSC, SSC, and IR regions in the *C*. *humilis* chloroplast genome with those of four other *Prunus* species.

In addition, we compared the SSC region and the IR-LSC and IR-SSC boundaries of *C*. *humilis* with other four species of *Prunus: P. maximowiczii, P. kansuensis, P. persica and P. pseudocerasus* (Fig 4). In the SSC region, all genes are conserved except tmS-AGA. The *rps19* gene is present at the LSC/IRA junction of the *C*. *humilis*, with a size of 142 bp which is shorter than other species’ *rps19* gene. The IR boundary regions also showed slight variations, which included gene deletions, insertions, expansions, and contractions (Fig 4). *C*. *humilis* and *P*. *maximowiczii* lost the *ycf1* gene in IRA/SSC boundary region. Moreover, *rps19* gene was deleted in *P*. *maximowiczii*, but inserted in *C*. *humilis* at the IRB/LSC boundary. The size of these bordering genes also varied. The small *ycf1* gene ranged in size from 1,003 bp to 1,059 bp, and that of the larger *ycf1* gene ranged from 5,607 bp to 5,664 bp, with the largest observed in *C*. *humilis*. At the IRB/LSC junction, the *rps19* gene was within the range of 183 bp–210 bp, whereas *P*. *maximowiczii* has no *rps19* gene at this area.

No major errors in genome assembly were observed in this study, the *ycf1* and *rps19* genes showed deletions, insertions, expansions and contractions among the five species, indicating that these genes may be used as markers in studying the evolution of the IR/SSC and IR/LSC junction in the *Prunus*.

### SSR analysis

**Fig 5:**
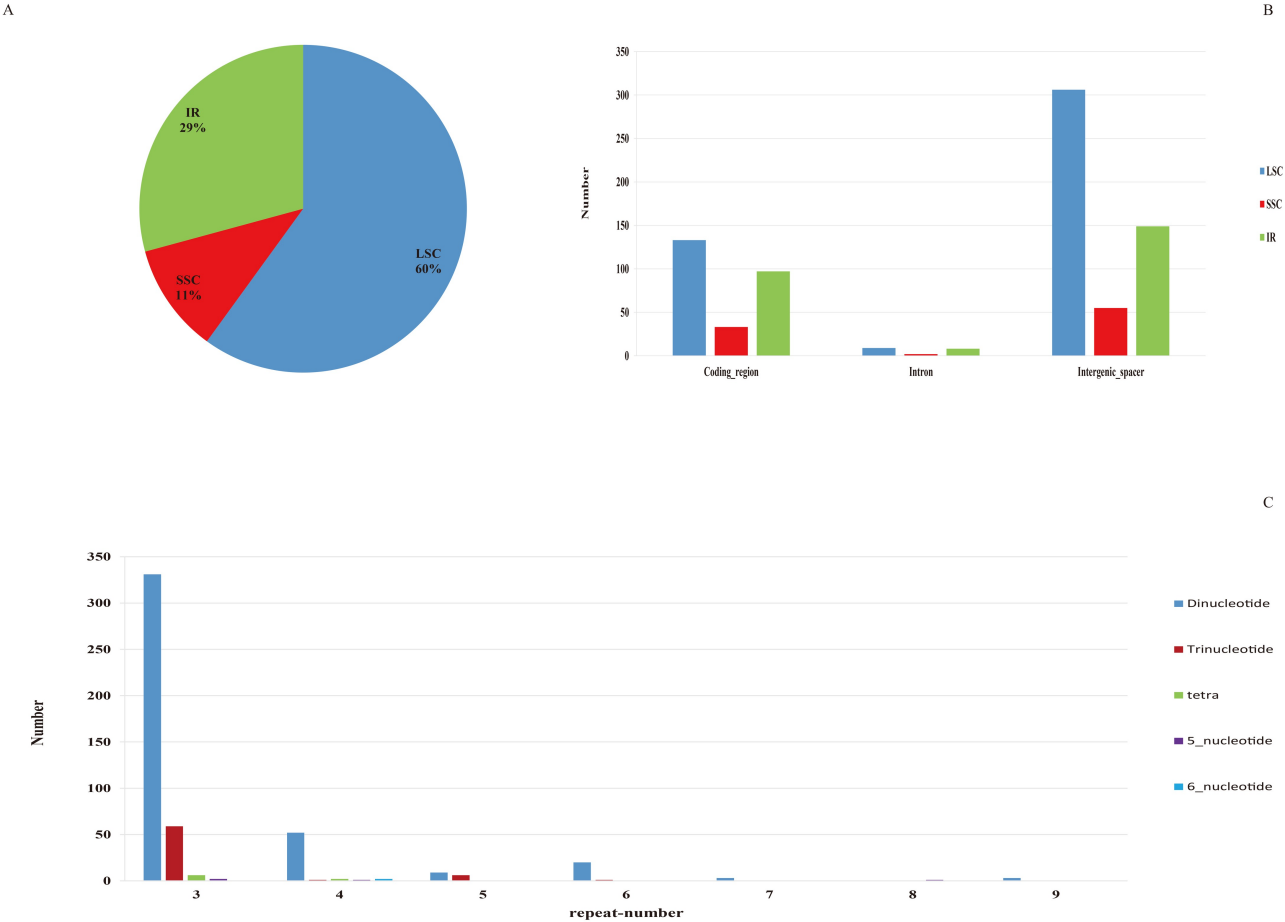
The distribution and type of SSRs in the chloroplast genome of *C*. *humilis*. (A) Percentage of SSRs in the LSC, SSC, and IR regions. (B) Distribution of SSRs in the protein-coding regions, introns, and intergenic spacers of the LSC, SSC, and IR regions. (C) Presence of polymers in the chloroplast genome of *C*. *humilis*.

A total of 510 SSRs were identified in the chloroplast genome of *C*. *humilis*. Then, we studied the distribution, presence, and types of SSRs (Fig 5). Of the 510 SSRs, 306 (60%), 55 (11%), and 149 (29%) were discovered within the LSC, SSC, and IR regions, respectively (Fig 5A). In addition, 263, 510, and 19 SSRs were situated within the protein-coding regions, intergenic spacer regions, and introns, respectively. The presence of SSRs in the SSC and IR regions was the same as that above except for the LSC, i.e., the SSR number in the protein-coding regions was the highest, followed by intergenic spacers, and then introns. Furthermore, the predominant SSRs included dinucleotides, trinucleotides, and tetranucleotides, with the repeat-number equal to three, and dinucleotide span the repeat-number range from 3 to 9.

### Phylogenetic analysis

**Fig 6:**
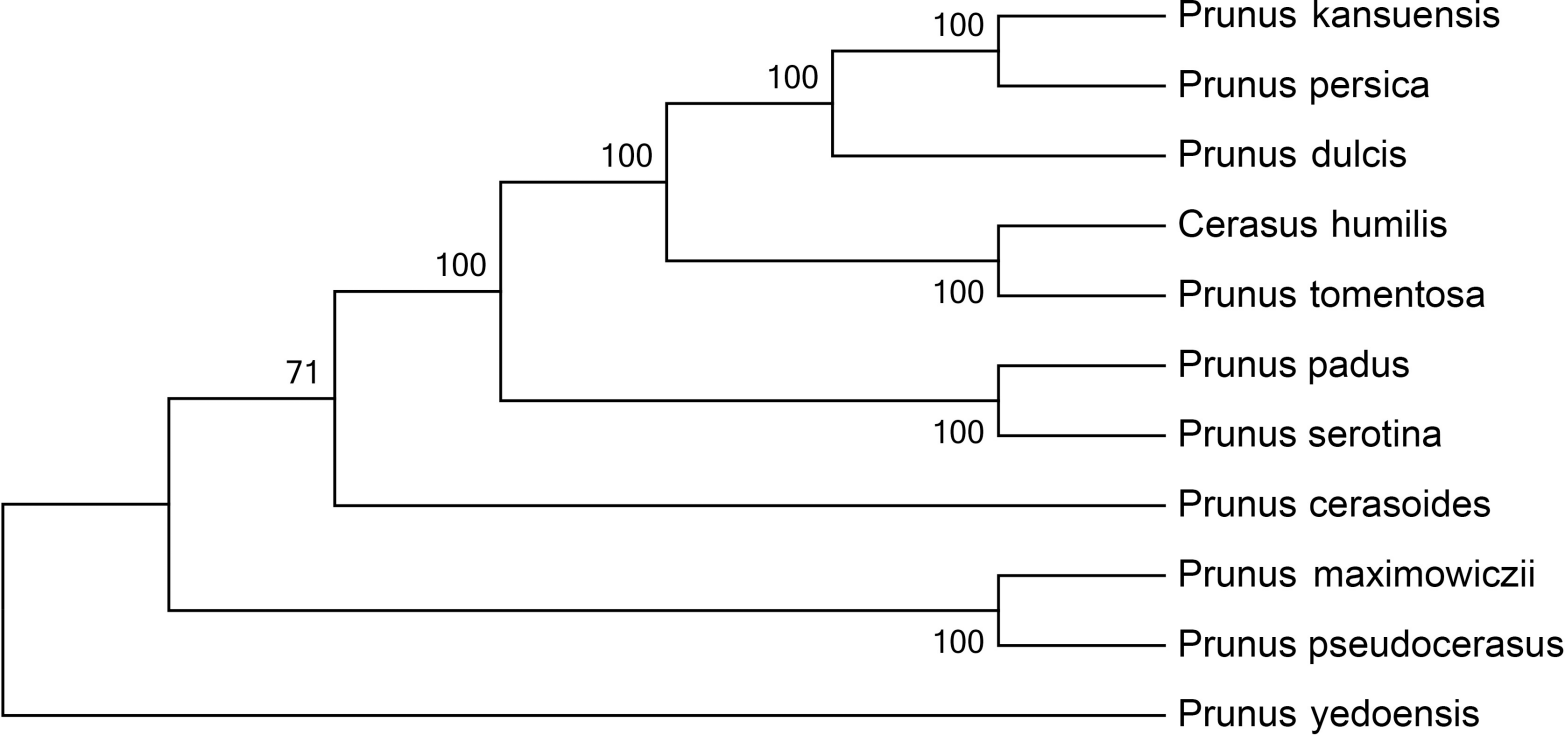
Phylogenetic reconstruction of the *Prunus* species based on 61 conserved coding-protein genes using the maximum likelihood approach.

The 61 conservative coding-protein genes were used to analyze the phylogenetic relationships among the members of *Prunus* (Fig 6). The phylogenetic tree demonstrated that *P*. *persica* and *P*. *kansuensis* formed a monophyletic clade while *C*. *humilis* and *P*. *tomentosa* formed another monophyletic clade. These two clades were then clustered with *P*. *dulcis* to form a larger branch (Fig 6). *C*. *humilis* and *P*. *tomentosa* were previously classified to the subgenus *Microcerasus* based on their morphological characteristics [14]. The phylogenetic results of this study provide new evidence for this classification at the genome level.

## Discussion

In this study, the complete chloroplast genome of *C*. *humilis* was sequenced and annotated, and the SSRs and comparative genomics of the genome were analyzed. Moreover, phylogenetic reconstruction of *C*. *humilis* and ten other *Prunus* species was performed. Although the chloroplast genomes are highly conserved in terms of genomic structure and size, the IR/SC border genes varied in size and in type, which is a typical feature of chloroplast genomes [10,26,27]. The genomic structure of ten *Prunus *species and *C*. *humilis* are also relatively conserved, whereas differences in the presence of genes in the IR/SC border region were observed. *P*. *kansuensis* and *C*. *humilis* lost the *ycf1* gene in the IRA/SSC border region. *Ycf1* is one of the giant ORFs in most higher plants of chloroplast genomes and it usually spans the boundary of the IR and SSC regions of the plastid genome [28]. However, in the orchid genus *Phalaenopsis*, the entire *ycf1* gene is situated within the SSC region [29,30]. In addition, the function of the *ycf1* gene remains elusive [31]. In tobacco, it has been suggested that the *ycf1* gene does not encode a functional protein while some other studies have indicated that it encodes a protein that is essential for cell survival or related to the ABC-transporters [31,32]. The higher variation in the *ycf1* gene could provide superior resolution and support at lower taxonomic levels in *Orchidaceae* [28]. In our study, the small size of the *ycf1* gene in IRA/SSC boundary ranges from 1,003 bp to 1,059 bp which is significantly smaller than the larger *ycf1* gene (size range: 5,607 bp-5,662 bp). The role of this gene deletion in the evolution of chloroplast genome of *Prunus* requires further investigations.

In this study, two copies of *C*. *humilis rps19* gene were found in the boundaries of IRA/LSC and IRB/SSC repectively, whereas the *rps19* gene was deleted from the IRB/SSC boundary in *P*. *kansuensis*. Similar results were found in two leguminous plants *Cajanus* and *Millettia* [33–35]. In addition, it was reported that one of the two *rps19* genes in the IR/SC boundaries is usually a pseudogene. For example, *Dianthus* encodes one copy of the *rps19* gene at the IRB/SSC junction and a pseudogene *rps19* at IRA/LSC junction while the size of the pseudogene *rps19* is shorter than that of the regular *rps19* gene [34]. In three *Cardiocrinum* (Liliaceae) species, the *rps19* gene located in the boundary between LSC and IRA apparently lost its protein-coding ability due to partial gene duplication [36]. The *rps19* gene of *C*. *humilis* located in the boundary between LSC/IRA is much shorter than those of other four *Prunus* species which means it could also be a pseudogene. Roles of the *rps19* gene’s insertion and deletion observed in the chloroplast genomes of *Prunus* species remains to be elucidated.

*C*. *humilis* belongs to the genus *Prunus* which consists of approximately 250 species. Many economically important fruit crops such as cherry, plum, apricot, almond, peach are in this genus. However, there are different opinions about how *C*. *humilis* should be grouped and named. A famous Chinese plant taxonomist earlier classified *C*. *humilis* as *Rosaceae cerasus* because he believed *C*. *humilis* is a close relative to *P*. *pseudocerasus* (cherry) [14]. However, the phylogenetic trees we built in this study showed that *C*. *humilis* is closer to *P*. *tomentosa* than to *P*. *pseudocerasus*. Our results agree with another classification which put *C*. *humilis* and *P*. *tomentosa* to the same *subgenus Microcerasus* based on their morphological characteristics [37]. Therefore, the results of this study might draw attentions of other scientists who have been working on clarifying the phylogenetic relationships among species of genus *Prunus*. More chloroplast genome sequences will be needed to improve our understanding of the evolution of *Prunus* species. Our findings not only provide the foundation for further studies on the evolution of chloroplast genome of *C*. *humilis* and *Prunus* species, but also serve as the basis for the molecular identification of *Prunus* species.

*C*. *humilis* was a wild species 30 years ago and it had sour and astringent taste which was quite different for people to eat. Our team started a program including domestication and breeding of this species in 1987 aiming at improving its fruit characters for commercial cultivation. One part of this program is to introduce specific advantageous traits into *C*. *humilis* from cultivated crops by crossing. However, little has been accomplished despite a lot of effort. Success in breeding is determined by genetic compatibility and chloroplast genomes serve as a valuable tool for identifying plants that are likely to be closely related and, therefore, genetically compatible [38]. We believe that chloroplast genome of *C*. *humilis* can provide useful information for us to select proper parental combinations to increase breeding efficiency. Another key application of the chloroplast genome of *C*. *humilis* is the identification of commercial cultivars and the determination of their purity. DNA barcodes could be developed from its chloroplast genome and be used to identify varieties and their offsprings.
